# A guide for the type A aortic dissection consultation and surgical planning: Key points for cardiac surgery trainees

**DOI:** 10.1016/j.xjon.2025.04.020

**Published:** 2025-05-08

**Authors:** Ryaan El-Andari, Michael C. Moon

**Affiliations:** Division of Cardiac Surgery, Department of Surgery, University of Alberta, Edmonton, Alberta, Canada

**Figure:**
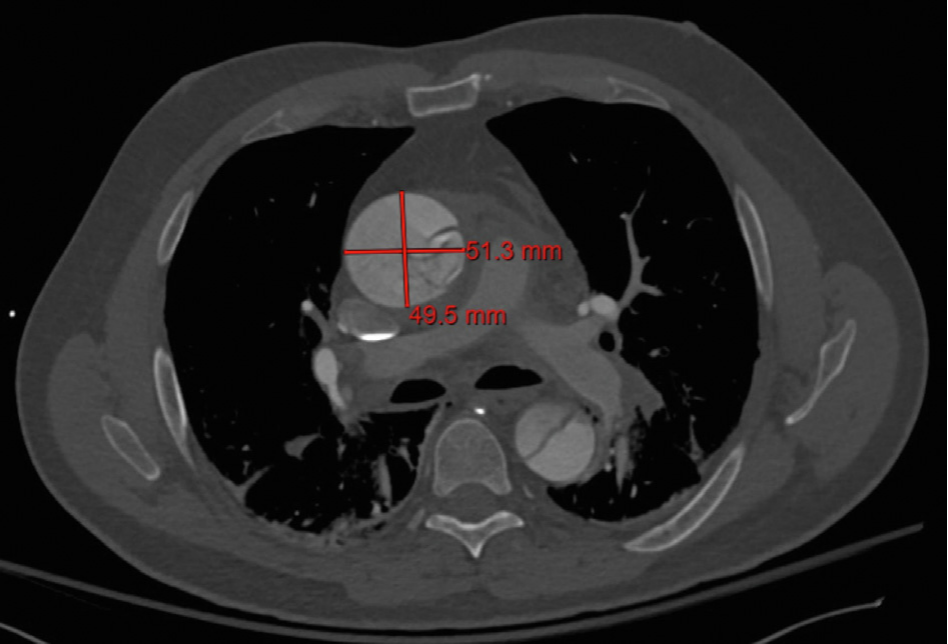
Computed tomography image with measurement of total aortic diameter.

**Central Message**

The ATAAD consultation requires a detailed yet efficient history and physical examination to collect the essential information for diagnosis and surgical planning.

**Perspective**

ATAAD is an emergency condition and among the most urgent cardiac surgery consultations. The on-call resident is often the first team member to see the patient. The ATAAD consultation requires specific information to guide management that must be performed in a timely manner given the acuity of the condition.

Acute type A aortic dissection (ATAAD) is an emergency and life-threatening condition. Once cardiac surgery is consulted, the on-call resident is often the first team member to see the patient and ATAAD is among the most urgent cardiac surgery consultations. Requiring emergency intervention to prevent mortality or significant morbidity secondary to progression of dissection or malperfusion, the ATAAD consultation must be performed in a timely manner but needs to collect numerous pieces of essential information that will guide management. Herein, we provide a guide to the aortic dissection consultation, reviewing key points in the workup and management of ATAAD.

## History and Physical Exam

Aortic dissection has often been called the great mimicker given its ability to present with a range of nonspecific symptoms—even without symptoms at times—making the initial diagnosis difficult.^1^ The differential diagnosis for patients with ATAAD is broad and is driven by the presenting symptoms. For example, patients presenting with chest pain may be experiencing myocardial infarction, pulmonary embolism, pneumothorax, or esophageal spasm. Patients presenting with acute neurologic dysfunction likely have cerebrovascular accident or seizure on their differential. Most often, cardiac surgery consultation is requested after the diagnosis is made on imaging, although ATAAD may present in other settings such as iatrogenic dissections caused by cardiac catheterization or cardiac surgery.

Symptoms at the time of presentation provide clues to the extent of dissection and branch vessel involvement. Classic aortic dissection-related pain is often described as sudden onset, severe, sharp or tearing, central chest pain, and radiating to the back.^1^, ^2^, ^3^ Chest pain can also be angina-like with dissection extension into and ischemia of the coronaries.^1^^,^^2^ Neurologic signs and symptoms of stroke suggest head vessel involvement or spinal cord ischemia; dyspnea suggests airway compression, heart failure, or tamponade; abdominal pain may indicate visceral involvement; elevated creatinine results from renal artery involvement; and peripheral weakness or numbness may suggest involvement of peripheral branch vessels.^1^, ^2^, ^3^

A thorough history is central to the evaluation of ATAAD and will inform the team of the patient's baseline function, past medical history (with a specific focus on contributing conditions such as hypertension, smoking history, and substance use), and family history (being sure to inquire about family history of sudden death, tall family members, and aneurysms) will help determine surgical risk and prognosis. Questions regarding baseline function may include inquiries to tasks required at home, such as grocery shopping, which require adequate mobility and executive function with respect to managing lists and money. These details provide insights into the patient's baseline function and expectations for postoperative recovery. The presence of connective tissue disorders, including Marfan syndrome, Ehlers-Danlos syndrome, or Loeys-Dietz syndrome, often change management, suggesting the need for more extensive surgical intervention.^1^, ^2^, ^3^ Other conditions of interest include Turner syndrome, vasculitis, pregnancy, or a history of valvular heart disease, particularly bicuspid aortic valves, which are important to note because they indicate etiology and risk factors. In cases where patients present with a reduced level of consciousness or have already been intubated, a history can still be obtained via thorough chart review and collateral history from the next of kin. A reduced level of consciousness may be an indication of cerebral malperfusion and would suggest to a surgeon that emergency intervention should be performed as soon as possible.

It is important to note the interventions that have been undertaken so far in the patient's clinical course. ATAAD is often misdiagnosed initially as a myocardial infarction or pulmonary embolism before definitive diagnosis. In these cases, patients may be given an anticoagulant or antiplatelet agents empirically and will inform the surgical team that hemostasis may be difficult to achieve. The anesthesia team may be able to prepare for this by administering reversal agents and ensuring coagulation factors are replaced where needed. Any anticoagulation or antiplatelet therapy should be stopped immediately upon diagnosis of ATAAD and should be reversed if possible either preoperatively or intraoperatively to reduce risk of postoperative bleeding.

**Figure 1:**
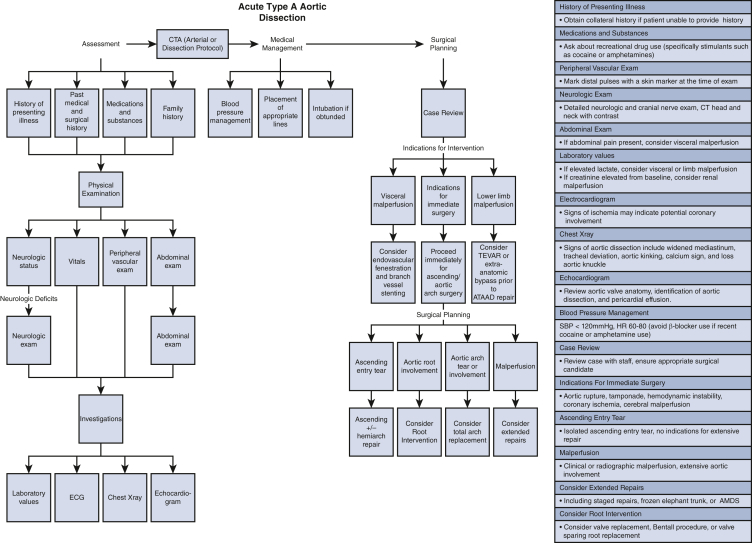
Detailed flow diagram for the type A aortic dissection consultation and management. *CT*, Computed tomography; *SBP*, systolic blood pressure; *HR*, heart rate.

**Figure E1:**
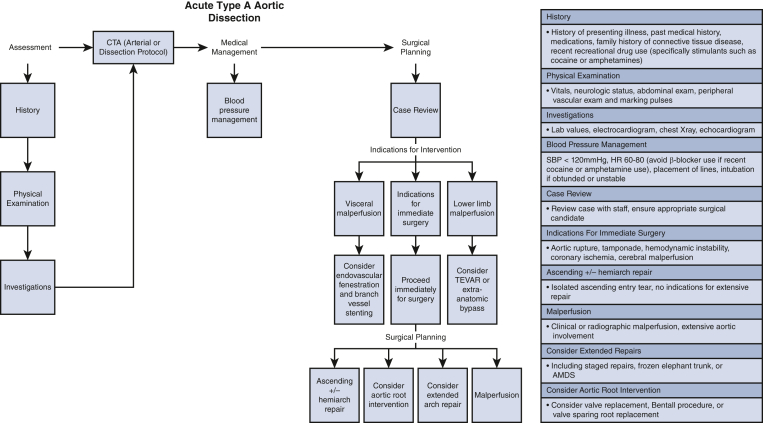
Simplified flow diagram for the type A aortic dissection consultation and management. *SBP*, Systolic blood pressure; *HR*, heart rate.

Several key points must be addressed during the physical exam. At first encounter with the patient, one must take note of his or her level of consciousness and appearance. Identification of early signs of stroke is essential. Four-limb blood pressures should be obtained as both a baseline and to identify any malperfused limbs. Signs that may suggest an aortic dissection include a widened pulse pressure, hypertension, hypotension, regurgitant heart murmur, or diminished peripheral pulses.^1^^,^^2^ A brief neurologic exam can identify deficits in motor or sensation functions in any of the cranial nerves or limbs. Examination of the abdomen helps with discerning whether the patient has experienced abdominal pain. Laboratory findings such as rising lactate level suggest visceral malperfusion and a rising creatinine level compared with baseline suggests renal malperfusion.^4^ Rising lactate levels may also be suggestive of limb malperfusion. Finally, palpation and marking of the distal and femoral pulses with a skin marker will be useful for intraoperative management and postoperative care (Figures 1 and E1). Limb malperfusion may present with a cool and pulseless limb with new motor weakness or sensory deficits.

## Investigations

Basic laboratory values are often unremarkable, although elevated creatinine level indicates renal ischemia, elevated lactate level suggests organ or limb ischemia, and elevated D-dimer level is common.^2^ Chest radiograph images are also unremarkable in many cases but may demonstrate a widened mediastinum, tracheal deviation, aortic kinking, double or irregular aortic contour, new pleural effusion, calcium sign (in which a separation of intimal calcification from the aortic wall is >5 mm), deviation of a nasogastric tube, or loss of the aortic knuckle.^1^ Electrocardiogram may be nonspecific but may show ST segment changes with coronary involvement resulting in myocardial ischemia suggesting dissection of the aortic root. Knowledge of a history of coronary artery disease can help to differentiate acute from chronic changes in this setting. A transthoracic echocardiogram may show a bicuspid aortic valve, which is more common in patients with ATAAD. Key details to note include new wall motion abnormalities with aortic root and coronary involvement, aortic valve insufficiency, and pericardial effusion that may suggest a contained aortic rupture and pending hemodynamic instability that will help guide the surgeon in surgical planning.^2^ In the case of aortic rupture, peripheral cannulation should be considered before sternotomy to allow for cooling and prevent life-threatening exsanguination on entering the chest by allowing for pump sucker bypass if there is an aortic rupture. A transesophageal echocardiogram is often performed intraoperatively and can provide additional information regarding aortic root involvement and aortic valve competence.

**Figure E2:**
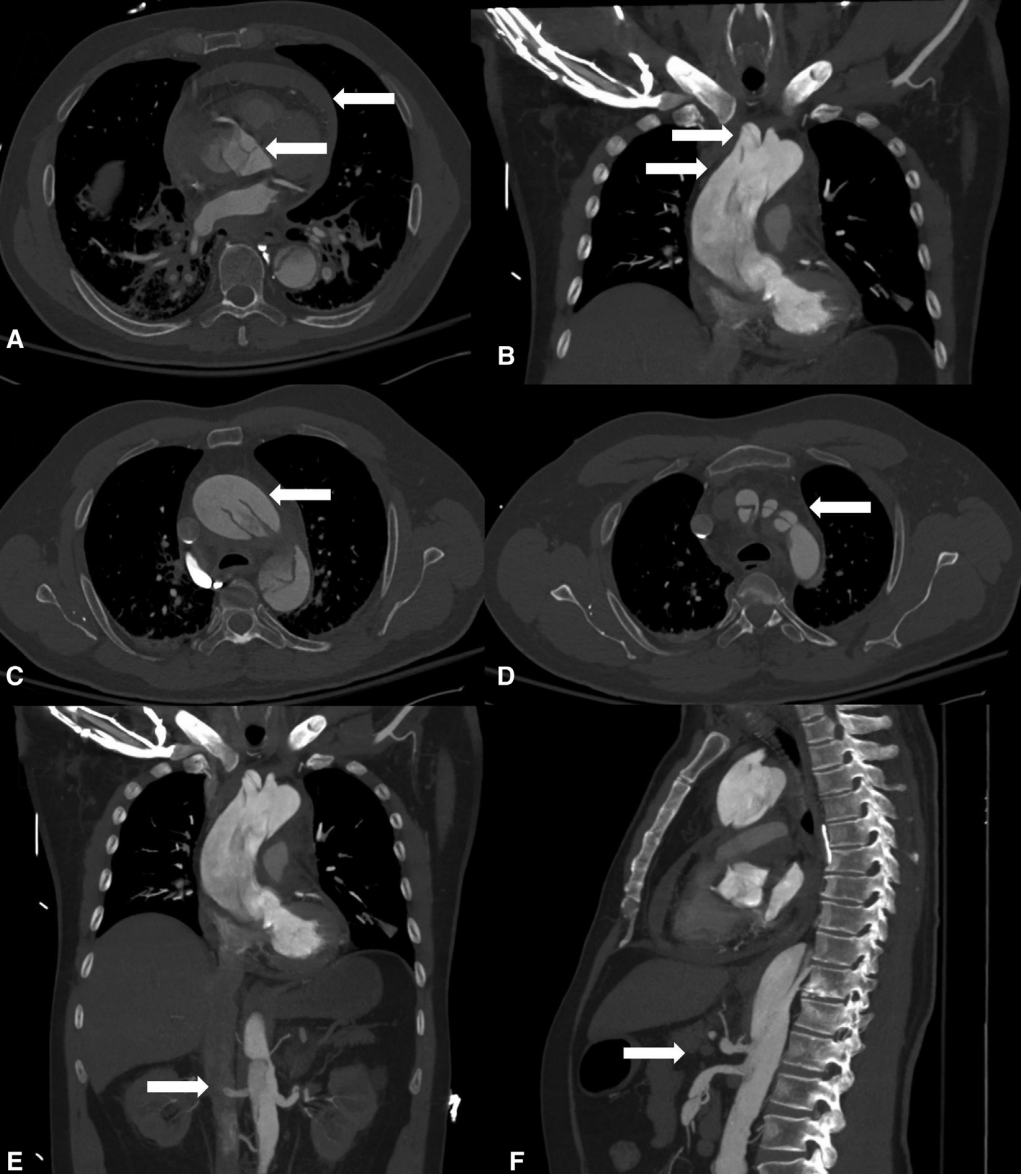
Preoperative computerized tomography scan images showing aortic dissection extending into the aortic root as well as hemopericardium (A); dissection of the ascending aorta, including disruptions between the true to false lumen suggesting an entry tear in the ascending aorta and dissection of the innominate artery (B); an entry tear in the ascending aorta (C); dissection of all 3 of the head vessels (D), perfusion of the right renal artery from the false lumen and the left renal artery from the true lumen (E), and dissection of the celiac and superior mesenteric arteries (F). The *arrow* is indicating the right renal artery.

Although aortic dissection may be identified incidentally on suboptimal scans, such as an echocardiogram or computed tomography (CT) pulmonary embolism protocol, the CT angiogram (CTA) scan of the chest, abdomen, and pelvis is the gold standard for the diagnosis of aortic dissection and surgical planning.^1^, ^2^, ^3^ Several key points are required to note during review of the CTA, an example of which can be found in Figure E2. First, the aortic dissection must be identified and classified. Whereas a handful of classification systems exist, the Stanford or Debakey classification systems are the ones that differentiate type A and B dissections and will guide management.^2^^,^^3^ Intramural hematoma and penetrating atherosclerotic ulcers are other types of acute aortic syndromes that may be identified on imaging and often require surgical repair if patients are symptomatic or at risk of dissection. Identification of ascending, arch, or descending aortic involvement will determine whether the patient needs immediate surgical intervention, endovascular repair, or medical management. The true lumen and false lumen should be identified. Although difficult to differentiate at times, the true lumen can be identified because it is often smaller being compressed by the higher-pressure false lumen, will have brighter contrast, may have calcifications, and can be traced back to the aortic root and left ventricular outflow tract. The extent of the dissection, including the most proximal and distal extents, aortic root or coronary involvement, and size of the aorta should be noted.

**Figure E3:**
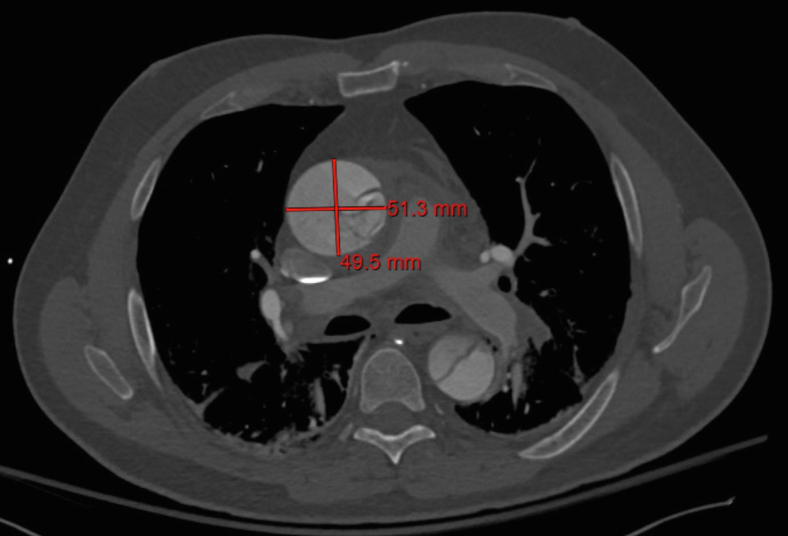
Illustration of measurements of the aorta on computed tomography CT scan. This involves orienting the image so that a cross section of the aorta is available that is perpendicular to blood flow. Measurements should then be taken from the inner edge to the inner edge of the aorta, or in cases of atherosclerotic disease or wall thickening from the outer edge to the outer edge, and ideally 2 measurements are taken perpendicular to each other. In the case of an oval shaped aorta, the first measurement should be in the longest axis and the second measurement perpendicular to the first.

Measurements are taken at multiple sites in the aorta, including the aortic root, ascending aorta, aortic arch, descending thoracic aorta, and abdominal aorta but specific sites of measurement will depend on the extent of disease. Measurements should be focused on areas of dissection, with measurements also taken in any uninvolved aorta proximally or distally to the dissection. In cases where measurements are not provided on the CT report, these are the minimum required measurements and should be feasible to obtain when viewing a CT on any standard image viewing software. Aortic arch measurements may be taken in a variety of ways, with consistency in technique for measurement being key. One approach to measurements of the aorta on a CT scan includes orienting the image so that a cross-section of the aorta is available that is perpendicular to blood flow. Measurements should then be taken from the inner edge to the inner edge of the aorta—or in cases of atherosclerotic disease or wall thickening from the outer edge to the outer edge—and ideally 2 measurements are taken perpendicular to each other. In the case of an oval-shaped aorta, the first measurement should be in the longest axis and the second measurement perpendicular to the first^1^ (Figure E3).

Any branch vessels that are dissected, including clear signs of organ malperfusion, are also of importance. Close inspection of the CTA should reveal the location of entry tears and re-entry tears identified by communications between the true and false lumens, seen as disruptions between the border dividing the 2. Additional useful information includes aortic anatomy, anomalous branches, or courses of the branch vessels. Artifacts on the CT scan may complicate the diagnosis. In cases of high likelihood based on clinical presentation, proceeding with surgery may still be indicated if ATAAD is suspected on imaging. In a stable patient where the likelihood based on clinical characteristics is low, the CT scan may be repeated to confirm diagnosis.

In summary, the key information required from the CT scan includes the extent of dissection, aortic measurements, location of entry tears, and branch vessel involvement. Once the above information is gathered, a surgical plan can be prepared (Table 1, Figures 1 and E1).

**Table 1:** Checklist for the type A aortic dissection consultation

History
Symptom onset, characterization, change over time
Past medical history
Past surgical history
History of recreational substance use
Caution with recent cocaine use and β-blockade given risk of unopposed α-agonism
History of connective tissue disorders
Family members with a diagnosis, aneurysms, hypermobile joints, height, sudden cardiac death
Neurologic symptoms including syncope, loss of bowel or bladder control, limb weakness, or paresthesia
Baseline mobility and function
Obtain collateral history (if possible)
Physical exam
Vital signs, basic physical exam
Neurologic status and neurologic exam if concern for neurologic deficits
Examine the chest for previous surgical incisions
4-limb blood pressure
Marking of distal pulses
Investigations
Basic labs
Complete blood count, electrolytes, creatinine, arterial blood gas
Electrocardiogram
Chest radiograph
Echocardiogram
Optional depending on urgency, can obtain intraoperative transesophageal echocardiogram
Computed tomography angiography
Arterial phase or dissection protocol; scan of the chest, abdomen, and pelvis

## Management

Medical management is required for all aortic dissections, although it should not delay emergency surgical intervention.^2^^,^^5^ Blood pressure control using intravenous β-blockers and pain management is imperative in the interim before surgical repair.^1^ Clarification of substance use, especially recent cocaine or amphetamine use, is important given the concomitant use of β-blockers will result in unopposed α-agonism. Unopposed α-agonism may occur in the setting of overstimulation of the adrenergic system through cocaine or amphetamine use. The administration of β-blockers (nonselective β-adrenergic antagonists) does not inhibit the α-agonists and results in unopposed α-agonism, which leads to severe hypertension and coronary ischemia.^6^

Several contraindications exist to immediately proceeding to the operating room for ATAAD. Contraindications for surgical repair must be identified early to prevent unnecessary invasive procedures. A discussion must be had with the patient when possible to ensure proceeding with surgery would be in line with the patient's wishes. In the case of an obtunded patient who is not able to provide informed consent, collateral history and consent can be obtained from the family or substitute decision-maker. Surgical risk must be determined preoperatively to identify patients with prohibitive preoperative surgical risk. A stroke causing severe neurological deficits, advanced age, and significant comorbidities may be relative contraindications for aortic dissection repair and surgical risk must be weighed with the benefits and chance of functional recovery. Comorbidities and frailty are essential to identify because a frail patient may have a low chance of meaningful postoperative recovery and certain comorbidities, such as metastatic malignancy or other end organ failure, may both increase surgical risk and limit a patient's life expectancy should they survive the initial surgical repair. In cases of cardiac arrest or with ongoing cardiopulmonary resuscitation at presentation, intervention may be futile. The information above must be compiled to determine the probability of survival and quality of life postsurgery.^3^

Once the initial assessment has been completed and the patient appears to be a candidate for surgical repair, several steps must be taken by the consulted physician. For residents or fellows, the attending surgeon should be immediately informed of the case. If it is decided that surgical repair is appropriate, the operating room, anesthesia team, perfusionist, and blood bank should be informed to expedite preparation of the operating room and begin preparation of blood products. Consent should be obtained from the patient or substitute decision-maker, and a note should be documented in the chart reporting the history, physical exam, and plan.

In cases where organ malperfusion is the immediately life-threatening issue, endovascular or open repair of the distal aorta to resolve malperfusion and stabilize the patient before proximal aortic repair has been described allowing for initial stabilization but does carry the risk of interim mortality.^1^^,^^7^^,^^8^

Planning for surgical management starts with the cannulation strategy and temperature. Often, peripheral cannulation with bypass and cooling is initiated before sternotomy, although central cannulation may also be performed with the Seldinger technique and guidance with transesophageal echocardiogram.^9^ Options for peripheral cannulation include the axillary artery (most commonly the right) and femoral artery and vein.^3^^,^^10^ A cutdown can be performed and the graft anastomosed to either artery for arterial cannulation, and percutaneous access can be achieved for either arterial or venous cannulation, depending on surgeon preference.^10^ The right axillary artery provides an excellent cannulation site, if feasible, because once the chest is open the innominate artery can be clamped allowing for antegrade cerebral perfusion during circulatory arrest and reduced risk of stroke.^1^ Selection of peripheral arterial cannulation site depends on extent of dissection as well as surgeon preference. Identification of dissected vessels will help guide cannulation because it is preferable to avoid dissected arteries. Temperature selection is of significant importance when planning an ATAAD repair. Lower temperatures provide greater cerebral, spinal, and organ protection facilitating longer circulatory arrest times to perform distal anastomoses although they are associated with worsening coagulopathy at the end of the case. Temperatures during circulatory arrest traditionally range from 18 to 28 °C and depend on surgeon preference and operative plan.

**Figure E4:**
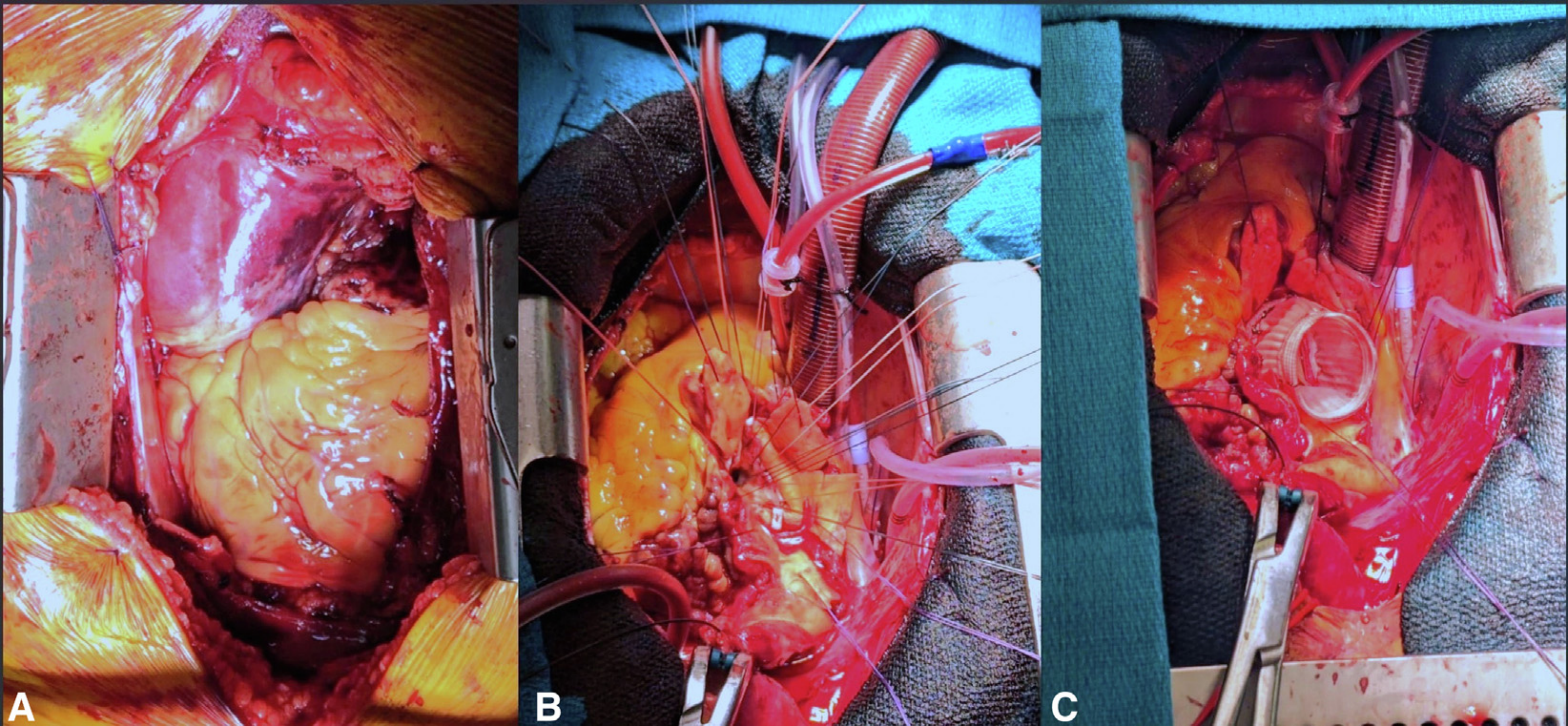
Intraoperative images of an acute type A aortic dissection repair, including preoperative appearance of the dissected ascending aorta (A), aortic root following resection of the aorta and placement of annular sutures (B), and aortic graft once the aortic root has been replaced (C).

The extent of surgical repair will depend on the extent of the dissection, in addition to the surgeon's experience. Beginning at the aortic root, dissection extending into this area may require a root intervention to restore aortic valve function and coronary perfusion (Figures 1, E1 and E4). If the aortic valve is compromised, repair or resuspension of the valve is the first choice to repair aortic insufficiency secondary to aortic dissection.^1^^,^^2^ If the valve is not amenable to repair, it must be replaced.^2^ A valved conduit may be used to complete a Bentall procedure. Where dissection extends into the aortic root without valve involvement, a valve-sparing root procedure may be beneficial.^1^^,^^2^ In the case of valve dysfunction where a root replacement is not required, an aortic valve replacement with ascending aortic replacement (Wheat procedure) may suffice.

Once into the aortic arch, generally, a hemiarch repair is the minimum required for a life-saving repair. Dissection into the arch does not necessarily require a total arch replacement.^10^ Patients with connective tissue disorders, large aneurysms, young patients, or the presence of entry tears in the arch may benefit from an arch procedure.^3^^,^^10^ Several options exist for an arch procedure. A total arch replacement, including reimplantation of the head vessels will resect any tears in the arch. The placement of an elephant trunk (ET) graft (free floating graft in the descending thoracic aorta) will provide a landing zone for future or concomitant endovascular procedures or allow clamping of the ET to sew a graft to the distal end. A frozen ET involves a total arch replacement with a stent deployed into the descending thoracic aorta with a proximal landing zone in the distal arch graft, addressing more distal disease in the descending thoracic aorta and stenting the true lumen open and there are several hybrid stent grafts available such as the E-Vita (Artivion) or Thoraflex (Terumo Aortic) or hybrid stent devices such as the AMDS Hybrid Prosthesis (Artivion).^2^ Caution must be exercised with covered stents because long segments of covered stents exclude the intercostal and lumbar arteries, increasing the risk of spinal ischemia. Dissected branch vessels resulting in malperfusion require careful consideration and may require intervention during aortic dissection repair or via endovascular approaches postoperatively.

## Conclusions

ATAAD is a dangerous and complex condition. In clinical settings, there is limited time from diagnosis to surgical intervention and a large volume of information must be obtained before proceeding to the operating room. A thorough but directed history, physical exam, and review of imaging are required for operative planning. The operative plan should look to save the patient's life and prevent future adverse events.

## Conflict of Interest Statement

Dr Moon receives consulting fees from Artivion. The other author reported no conflicts of interest.

The *Journal* policy requires editors and reviewers to disclose conflicts of interest and to decline handling or reviewing manuscripts for which they may have a conflict of interest. The editors and reviewers of this article have no conflicts of interest.
